# Prevalence of Tinea Capitis among School Children in Nok Community of Kaduna State, Nigeria

**DOI:** 10.1155/2016/9601717

**Published:** 2016-07-04

**Authors:** Josephine Dogo, Seniyat Larai Afegbua, Edward Christopher Dung

**Affiliations:** ^1^Department of Microbiology, Ahmadu Bello University, Zaria, Kaduna State, Nigeria; ^2^Department of Dermatophilosis, National Veterinary Research Institute, Vom, Plateau State, Nigeria

## Abstract

In recent years, the prevalence of tinea capitis, an infection of the scalp by dermatophytes, has increased in children worldwide. This cross-sectional study was carried out to determine the prevalence and risk factor of tinea capitis among school children in Nok community of Kaduna State, Nigeria. A total of 100 children were screened and 45% were diagnosed to have tinea capitis after fungal culture and microscopy. The prevalence of tinea capitis among girls was higher (51.4%) than that among boys (41.5%) but not significantly different (*p* = 0.402). The prevalence with respect to age was lower for the age group 5–10 years (42.6%) than that of 11–15 years (50%) but was not significantly different (*p* = 0.524).* Trichophyton rubrum* (28.8%) and* Microsporum canis* (22.7%) were the most prevalent dermatophytes isolated and the least were* Trichophyton verrucosum* (4.5%) and* Trichophyton tonsurans* (4.5%). There were 73.3% single infection while 26.7% had 2–4 dermatophytes of the genera* Microsporum* and* Trichophyton*. The predisposing factors with statistically significant association with tinea capitis were number of children in the family (*p* = 0.02) and sharing of the same bed (*p* = 0.002). This indicates the high tendencies of spread of tinea capitis through human-to-human mode of transmission and possible animal contact. Community health education on the cause, mode of transmission, prevention, and prompt treatment of tinea capitis is recommended.

## 1. Introduction

Dermatophytosis refers to mycotic infections caused by a group of fungi that possess the propensity to colonize and infect superficial layers of the skin, hair, or nails [1]. This group of fungi that invade keratinized tissue associated with the stratum corneum of the skin, hair, and nails on the living host are called dermatophytes [1]. Dermatophytosis is caused by a group of fungi with predilection to colonize and infect stratum corneum of the skin, hair, and nails on the living host. Such fungi referred to as dermatophytes include members of the genera* Epidermophyton, Microsporum*, and* Trichophyton* [1]. Dermatophytes are grouped as either anthropophilic (e.g.,* Trichophyton violaceum, Trichophyton rubrum, *and* Microsporum audouinii*), geophilic (e.g.,* Microsporum gypseum* and* Microsporum nanum*), or zoophilic (e.g.,* Trichophyton mentagrophytes, Trichophyton verrucosum*, and* Microsporum canis*) based on their ecological niches; however, some dermatophytes are not strictly restricted to a particular ecological niche [1, 2].

Dermatophytosis has a worldwide distribution but is endemic to tropical regions as the growth of dermatophytes is facilitated by the warm and moist conditions [1, 3, 4]. Apart from climate, the variability in distribution of dermatophytes worldwide is attributed to other factors such as population migration patterns, lifestyle, primary host range, secondary host immunity, presence of immunodeficiency diseases, and patients attitude to prompt treatment following clinical presentation and standard of living [1, 5].

Depending on the body site affected, dermatophytosis may result in different clinical syndromes such as tinea capitis (hair shaft and scalp), tinea corporis (body), tinea cruris (groin), tinea pedis (foot), and tinea unguium (hand). Tinea capitis is an infection of the scalp and hair shaft caused by several species of* Trichophyton* and* Microsporum* [1, 6–8]. It is the most common dermatophytosis in children aged between six months and prepubertal age [8–11]. Tinea capitis is reported more in males than in females within prepubertal age [7, 12].

Tinea capitis is quite common in Africa and its prevalence among children is 14–86% [1]. Studies carried out in different parts of Nigeria have proven that causative agents of tinea capitis vary from one location to another. Nweze [13] reported* Trichophyton schoenleinii* as the predominant cause of tinea capitis in Borno. In a similar study carried out on 2150 itinerant Quranic scholars in Kano State,* Trichophyton rubrum* was the most prevalent followed by* Microsporum audouinii* [14]. Ayanlowo et al. [8] reported* Trichophyton mentagrophytes* as the most prevalent causative agent in a community in the southwestern part of Nigeria. Nweze and Okafor [15] reported* Trichophyton tonsurans* as the most prevalent aetiological agent of tinea capitis in Anambra State, Nigeria.

Studies are required to obtain information on its epidemiology and familial, social, and geographical factors [16, 17]. Although there are studies on prevalence of tinea capitis in different parts of Nigeria, there are no reports on epidemiologic studies on tinea capitis among school children in Kaduna State. Nok community, a rural settlement in Jaba local government area of Kaduna State, was selected for this study. This study was designed to determine the prevalence of tinea capitis and identify factors associated with the infection among primary school children in Nok community, Kaduna State, Nigeria.

## 2. Methods

### 2.1. Study Area

**Figure 1:**
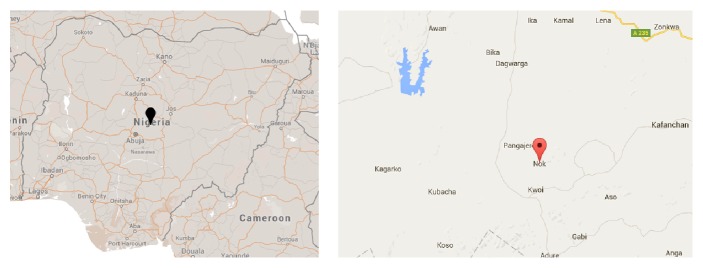
Maps showing Nok village in Kaduna State, Nigeria (taken from Google Maps).

The study was conducted in Nok community, a rural settlement located in Jaba local government area of Kaduna State, Nigeria (Figure 1). Following the accidental unearthing of Nok terracotta heads (dated to 500 BC) by tin miners in 1943, Nok is one of the most ancient sites of ironwork and terracotta figurine production in Africa [18]. The native language of the people is “*hyam.*” The occupations of the people include farming, trading, and mining. Nok is a typical African settlement with residents having large and extended families living together. The social amenities in the community include electricity, pipe borne water, community health centre, museum, police station, and government primary and secondary schools. Children in the community are enrolled in either of the two universal basic education primary schools.

### 2.2. Study Population

Ethical approval was obtained from the Kaduna State Ministry of Health and Local Authority of the Universal Basic Education of Jaba local government area, before the commencement of field work. The parents of only one hundred children gave consent for their participation in the study.

### 2.3. Community Education on Tinea Capitis

Parents and children were enlightened on tinea capitis, purpose of the study, and sample collection procedure in the local* hyam* language. Information such as the local name for tinea capitis, cause of tinea capitis, and local treatment method was obtained from the people in the community.

### 2.4. Sample Collection and Questionnaire Administration

Each child was examined in a well-lit room and the scalp was examined for scaly grey patches, lusterless hair strands, and purulent lesions [19]. The site of infection was cleaned with 70% alcohol and followed by the collection of scalp scrapings from actively growing margins of the lesions using sterile scalpel blades [8]. The samples were transported to the laboratory for culture and identification.

Questionnaires were designed and administered to obtain sociodemographic data such as age, gender, number of children in the family, and information on the sharing of fomites.

### 2.5. Culture, Identification, and Characterization of Dermatophytes

The scalp scrapings were cultured on Sabouraud's dextrose agar plates with 0.05 g chloramphenicol and 5% NaCl [20, 21]. The media were prepared according to the manufacturers' instructions and followed by the addition of sodium chloride and 0.05 g chloramphenicol as described by Weitzman and Summerbell [20]. The samples were inoculated onto the prepared medium and incubated for 3-4 weeks at room temperature and examined for growth weekly [22]. A thin preparation of the fungal culture was made with a drop of lactophenol cotton blue solution on a glass slide, covered with a coverslip and observed under a microscope. Dermatophytes were identified based on macroscopic (growth characteristics and pigmentation) and microscopic morphology (formation of macroconidia and microconidia or other typical elements) as described by David et al. [21].

### 2.6. Data Analysis

Data were analysed by *χ* ^2^ test for association using SPSS Statistics 18.0 (SPSS Inc., Chicago, IL, USA), with *p* < 0.05 defined as the minimum level of significance to test factors associated with tinea capitis.

## 3. Results

### 3.1. Level of Awareness on Tinea Capitis in Nok Community

The common name for tinea capitis in Nok community is* “shaak shantaan”* meaning “spider web.” The locals believe that tinea capitis is caused by spiders which urinate on the heads of the children, lay their eggs, and spin their web on the head; hence, this causes the radial spread of the infection. As, at the time of sample collection, the locals did not have any traditional form of treatment for tinea capitis infection, an approach taken in the management of lice is employed for the management of tinea capitis. In serious cases of tinea capitis, the child's hair is shaved to allow the thorough scrubbing of the scalp. Also, his/her clothings are soaked in hot water before washing and the sharing of fomites are avoided.

At the time of the study, the community had only one barbing salon. An interview with salon manager revealed the use of single hair clipper which is treated by spraying methylated spirit on the clipper and using a lighter to flame the clipper after every haircut.

### 3.2. Sociodemographic Characteristics of Study Subjects

A total of 100 scalp scrapings were collected and analysed for dermatophytes. The characteristics of the study participants are shown in Table 1. The age range of the study participants was 5–15 years. Of the 100 participants, 65% (65/100) were male and 35% (35/100) were female, giving a gender ratio of 1.86 : 1 (males : females). 68% and 32% of the participants were of the age range 5–10 and 11–15 years, respectively. 74% of the study participants had a family size of 1–5 members and 26% of the participants had a family size of 6–10 members. The average number of children per family was 4.3.

### 3.3. Prevalence of Tinea Capitis

After the incubation period and examining the microscopic and macroscopic features of fungal growth, there were 45 cases of dermatophytosis of which 33 cases and 12 cases had single and mixed dermatophytes, respectively. The overall prevalence of tinea capitis in the study population is 45%. Tinea capitis was more prevalent among females (51.4%; 18/35) than in males (41.5%; 27/65). However, the gender ratio in terms of tinea capitis was 1 : 1.3 (male : female) and did not differ significantly (*p* = 0.402). The prevalence of tinea capitis with respect to age was lower for the age group 5–10 years (42.6%; 29/68) than that of 11–15 years (50%; 16/32) but was not significantly different (*p* = 0.524). There was an association between tinea capitis and the number of children in a family (*p* = 0.02) and sharing of bed (*p* = 0.002) (Table 1). There was no association between tinea capitis and variables such as sharing of combs (*p* = 0.188), sharing of caps/scarves (*p* = 0.107), sharing of towels, hair cut at a barbing salon (*p* = 0.28), keeping and playing with pets (*p* = 1.00), and carrying objects on a bare head (*p* = 1.00).

### 3.4. Dermatophytes Frequency

Of the 45 cases, a total of 66 dermatophytes were isolated belonging to the genera* Microsporum* and* Trichophyton* (Table 2). The most frequently isolated dermatophyte was* Trichophyton rubrum* (28.8%; 19/66), followed by* Microsporum canis* (22.7%; 15/66), and the least were* Trichophyton verrucosum* (4.5%; 3/66) and* Trichophyton tonsurans* (4.5%; 3/66). Although 33 cases (73.3%) had single infections with dermatophytes, mixed infections with 2–4 dermatophytes of the genera* Microsporum* and* Trichophyton* were observed in 12 cases (26.7%). This is presented in relation to age group and gender in Table 3. 75% (9/12) of the mixed infections were with* Microsporum* and* Trichophyton* species, 16.7% (2/12) were with* Microsporum* species alone, and 8.3% (1/12) was with* Trichophyton* species alone.

## 4. Discussion

The high prevalence of tinea capitis (45%) is close to the prevalence of 43.53% obtained in a study by Akinboro et al. [19] in Osogbo, southwestern Nigeria. However, the prevalence of this study was higher than those obtained in other studies: 9.5% for study carried out on 2150 Quranic scholars in Kano State [14], 15.4% for a study on 604 children in a rural settlement in southwestern Nigeria [8], and 9.4% for a study in Anambra State involving 47723 children [23]. Moto et al. [24] recently reported a higher prevalence of 81.2% (122/150) among school children in Mathare, an informal settlement in Nairobi, Kenya. A number of factors may be attributed to the varying prevalence and frequency of tinea capitis in different parts of Nigeria as well as other countries [22]. These include population growth, close contact among infected children at home and school, and poor personal hygiene [24].

With respect to gender, prevalence was higher among females than males, but the association was not significant (*p* = 0.402). This is supported by the findings of some previous studies that reported higher infections among females in Nigeria [25] and Egypt [26].

Furthermore, it was observed that the prevalence of tinea capitis in prepubertal females (56.7%; 17/30) was higher than that of the males (35.3%; 12/34) while the prevalence of tinea capitis among the pubertal age range of 11–15 years was higher in males (48.4%; 15/31) compared to females (20%; 1/5). This is contrary to the findings of Sidat et al. [12] and Balci et al. [7]. This is contrary to some findings and the suggestion that scalp infection in females is less due to steroid-mediated inhibition of dermatophyte growth by progesterone and other similar compounds while males may be predisposed to scalp infection due to prepubertal factors such as level of fungistatic fatty acids [23].

Various conflicting views exist regarding the sexual predominance of tinea capitis which may be attributed to hair dressing and styling practices such as tight hair braiding, shaving of the scalp, plaiting, and the use of hair oils which may promote disease transmission. However, the precise role of such practices remains a subject of study [3, 27, 28].

The most prevalent dermatophyte isolated was the anthropophilic dermatophyte,* Trichophyton rubrum*. Other anthropophilic dermatophytes isolated were* T. tonsurans, T. schoenleinii, T. rubrum, *and* M. audouinii*. Zoophilic dermatophytes isolated were* T. verrucosum *and* M. canis *with* M. gypseum* as the only geophilic dermatophyte isolated. This finding is similar to that of a study carried out in Kano State, Nigeria, by Adeleke et al. [14], where* Trichophyton rubrum* (50.2%) was the most prevalent dermatophyte isolated. Emele and Oyeka [23] reported* Microsporum audouinii* as the most prevalent dermatophyte (42%), followed by* M. ferrugineum* (17%) and* Trichophyton mentagrophytes* (16%) following a study among school children in Anambra State, Nigeria. An aetiological shift with regard to the predominant causative agents of tinea capitis has been reported in different parts of the world including the United States, Germany, and parts of Nigeria. This is attributed to factors including the spread of dermatophyte by migrants, change in animal husbandry practices, climate change, and evolution of new genotypes [23].

The finding of 73.3% single infection and 26.7% mixed infection with* Trichophyton* and* Microsporum* species is supported by 56% single infection and 38% mixed infection reported by Moto et al. [24]. However, they reported* Epidermophyton* species apart from* Trichophyton* and* Microsporum* species. They attributed mixed infections to factors such as poor personal hygiene and inadequate treatment. Inadequate treatment may be supported by the data on revealed previous treatment of tinea capitis by 43.2% of those who had tinea capitis at the time of the study. This is contrary to the finding of Emele and Oyeka [23] and Grover et al. [28] who reported single-dermatophyte infections.

The dominance of anthropophilic dermatophytes may reflect important risk factors involved in the spread of tinea capitis in the community such as direct contact with infected humans or indirect contact with infected fomites [29]. This also may explain the significant association between tinea capitis and the family size and sharing beds. Although 94% of the children had pets or domestic animals at home and 6% did not have any pets or domestic animals, there was no significant association with the tinea capitis. Animals such as cats, dogs, rodents, pigs, chickens, cattle, and horses are sources of zoophilic dermatophytes, most often seen on exposed body sites [30, 31]. Data regarding the type of animals present in the homes was not collected during the present study.

## 5. Conclusion 

Tinea capitis had a prevalence of 45% among school children in Nok community, Kaduna State, Nigeria. This study showed that the most prevalent causative agents of tinea capitis in Nok community are the anthropophilic dermatophytes,* Trichophyton rubrum* (28.8%), followed by the zoophilic dermatophyte,* Microsporum canis* (22.7%). Other dermatophytes isolated were* Trichophyton mentagrophytes, Trichophyton tonsurans, Trichophyton schoenleinii, Trichophyton verrucosum, Microsporum audouinii*, and* Microsporum gypseum*. These dermatophytes were associated with single and mixed infections, except* Trichophyton mentagrophytes*, which was not isolated from the cases with mixed infection. There was an association between tinea capitis and the number of family members and sharing of the same bed. This may be attributed to the socioeconomic status of parents and living and sanitary conditions. Hence there is a need to create awareness on tinea infections and preventive measures (such as good personal hygiene and sanitary practice and prompt treatment). There is also a need to educate barbers on the proper disinfection of clippers and the importance of adhering to the procedures. Further studies in different parts of the country would be useful in understanding the epidemiological pattern and management of tinea capitis.

## Figures and Tables

**Table 1:** Demographic data and other factors in relation to tinea capitis among school children in Nok community, Kaduna State, Nigeria.

Factor	Subcategory	Number of children with tinea capitis	Total number of children	*p* value
Gender	Male	27 (41.5)	65	0.402
	Female	18 (51.4)	35	

Age group (years)	5–10	29 (45.3)	64	0.524
	11–15	16 (44.4)	36	

Family size	1–5	28 (37.8)	74	0.022^*∗*^
	5–10	17 (65.4)	26	

Father's level of education	None	2 (66.7)	3	0.100
	Primary	3 (30.0)	10	
	Secondary	31 (41.3)	75	
	Tertiary	9 (75.0)	12	

Mother's level of education	None	1 (100.0)	1	0.414
	Primary	12 (38.7)	31	
	Secondary	28 (45.2)	62	
	Tertiary	4 (66.7)	6	

Awareness about tinea capitis	Yes	35 (47.3)	74	0.497
	No	10 (38.5)	26	

Hair cut at barbing salons	Yes	19 (46.3)	41	0.280
	No	12 (35.3)	34	
	No response	14 (56.0)	25	

Sharing beds with other person(s)	Yes	36 (57.1)	63	0.002^*∗*^
	No	9 (24.3)	37	

Sharing combs with other person(s)	Yes	38 (48.1)	79	0.188
	No	6 (30.0)	20	
	No response	1 (100.0)	1	

Sharing caps or scarves with other person(s)	Yes	23 (54.8)	42	0.107
	No	22 (37.9)	58	

Sharing towels with other person(s)	Yes	33 (47.1)	70	0.661
	No	12 (40.0)	30	

Presence of domestic animals at home	Yes	42 (44.7)	94	1.00
	No	3 (50.0)	6	

Carrying objects on bare head	Yes	41 (45.6)	90	1.00
	No	4 (40)	10	

Previous treatment	Yes	16 (43.2)	37	0.624
	No	29 (46.8)	62	
	No response	0 (0)	1	

^*∗*^Significantly different (*p* < 0.05 defined as the minimum level of significance).

**Table 2:** Frequency of isolated dermatophytes from school children in Nok community, Kaduna State.

Dermatophyte isolated	Frequency	Percentage (%)
*Microsporum canis*	15	22.7
*Microsporum audouinii*	7	10.6
*Microsporum gypseum*	8	12.1
*Trichophyton rubrum*	19	28.8
*Trichophyton mentagrophytes*	4	6.1
*Trichophyton verrucosum*	3	4.5
*Trichophyton tonsurans*	3	4.5
*Trichophyton schoenleinii*	7	10.6

**Table 3:** Mixed dermatophyte infection among school children in Nok community, Kaduna State, with respect to age and gender.

Case code	Sex	Age group	Dermatophytes isolated
NK7	M	5–10	*M. canis, T. rubrum*
NK15	F	5–10	*M. gypseum, T. rubrum*
NK32	M	5–10	*T. tonsurans, T. schoenleinii, T. rubrum*
NK47	F	5–10	*M. canis, M. audouinii*
NK51	M	11–15	*T. verrucosum, M. canis*
NK67	M	5–10	*T. schoenleinii, M. canis, M. gypseum*
NK69	F	5–10	*T. rubrum, M. canis*
NK72	M	5–10	*T. schoenleinii, T. rubrum, M. canis, M. gypseum*
NK85	M	11–15	*T. schoenleinii, T. rubrum, M. canis*
NK86	M	11–15	*M. canis, M. audouinii*
NK90	M	11–15	*M. audouinii, T. rubrum, T. tonsurans*
NK94	F	5–10	*T. schoenleinii, T. rubrum, M. canis, M. gypseum*

M: male; F: female.
